# Large-scale Genome Analyses Provide Insights into Hymenoptera Evolution

**DOI:** 10.1093/molbev/msaf221

**Published:** 2025-09-12

**Authors:** Chun He, Yi Yang, Xianxin Zhao, Junjie Li, Yuting Cai, Lijia Peng, Yuanyuan Liu, Shijiao Xiong, Yang Mei, Zhichao Yan, Jiale Wang, Shan Xiao, Ziwen Teng, Xueke Gao, Hui Xue, Qi Fang, Gongyin Ye, Xinhai Ye

**Affiliations:** State Key Laboratory of Rice Biology & Ministry of Agricultural and Rural Affairs Key Laboratory of Molecular Biology of Crop Pathogens and Insects, Institute of Insect Sciences, Zhejiang University, Hangzhou 310058, China; State Key Laboratory of Rice Biology & Ministry of Agricultural and Rural Affairs Key Laboratory of Molecular Biology of Crop Pathogens and Insects, Institute of Insect Sciences, Zhejiang University, Hangzhou 310058, China; State Key Laboratory of Rice Biology & Ministry of Agricultural and Rural Affairs Key Laboratory of Molecular Biology of Crop Pathogens and Insects, Institute of Insect Sciences, Zhejiang University, Hangzhou 310058, China; State Key Laboratory of Rice Biology & Ministry of Agricultural and Rural Affairs Key Laboratory of Molecular Biology of Crop Pathogens and Insects, Institute of Insect Sciences, Zhejiang University, Hangzhou 310058, China; State Key Laboratory of Rice Biology & Ministry of Agricultural and Rural Affairs Key Laboratory of Molecular Biology of Crop Pathogens and Insects, Institute of Insect Sciences, Zhejiang University, Hangzhou 310058, China; State Key Laboratory of Rice Biology & Ministry of Agricultural and Rural Affairs Key Laboratory of Molecular Biology of Crop Pathogens and Insects, Institute of Insect Sciences, Zhejiang University, Hangzhou 310058, China; State Key Laboratory of Rice Biology & Ministry of Agricultural and Rural Affairs Key Laboratory of Molecular Biology of Crop Pathogens and Insects, Institute of Insect Sciences, Zhejiang University, Hangzhou 310058, China; Xianghu Laboratory, Hangzhou 311231, China; State Key Laboratory of Rice Biology & Ministry of Agricultural and Rural Affairs Key Laboratory of Molecular Biology of Crop Pathogens and Insects, Institute of Insect Sciences, Zhejiang University, Hangzhou 310058, China; Department of Entomology, Nanjing Agricultural University, Nanjing 210095, China; Institute of Quality Standard and Monitoring Technology for Agro-Products of Guangdong Academy of Agricultural Sciences, Guangzhou 510640, China; Ningbo Academy of Agricultural Sciences, Ningbo 315100, China; College of Plant Health & Medicine, Qingdao Agricultural University, Qingdao 266109, China; State Key Laboratory of Cotton Bio-breeding and Integrated Utilization, Institute of Cotton Research, Chinese Academy of Agricultural Sciences, Anyang 455000, China; State Key Laboratory of Cotton Bio-breeding and Integrated Utilization, Institute of Cotton Research, Chinese Academy of Agricultural Sciences, Anyang 455000, China; State Key Laboratory of Rice Biology & Ministry of Agricultural and Rural Affairs Key Laboratory of Molecular Biology of Crop Pathogens and Insects, Institute of Insect Sciences, Zhejiang University, Hangzhou 310058, China; State Key Laboratory of Rice Biology & Ministry of Agricultural and Rural Affairs Key Laboratory of Molecular Biology of Crop Pathogens and Insects, Institute of Insect Sciences, Zhejiang University, Hangzhou 310058, China; College of Advanced Agriculture Sciences, Zhejiang A&F University, Hangzhou 311300, China

**Keywords:** Hymenoptera, evolution, genome

## Abstract

The order Hymenoptera includes a large number of species with diverse lifestyles and is known for its significant contributions to natural ecosystems. To better understand the evolution of this diverse order, we performed large-scale comparative genomics on 131 species from 13 superfamilies, covering most representative groups. We used these genomes to reveal an overall pattern of genomic change in terms of gene content and evolutionary rate throughout hymenopteran history. We identified genes that possibly contributed to the evolution of several key innovations, such as parasitoidism, wasp-waist, stinger, and secondary phytophagy. We also discovered the distinct genomic trajectories between the clade containing major parasitoid wasps (Parasitoida) and stinging species (Aculeata) since their divergence, which are involved in many aspects of genomic change, such as rapidly evolving gene families, gene gain and loss, and metabolic pathway evolution. In addition, we explored the genomic features accompanying the three independent evolution of secondary phytophagy. Our work provides insights for understanding genome evolution and the genomic basis of diversification in Hymenoptera.

## Introduction

Hymenoptera is a diverse order of insects, which includes more than 153,000 described species, such as sawflies, wasps, ants, and bees (Grimaldi and Engel 2005; Huber 2017; Peters et al. 2017). These hymenopterans play a pivotal role in natural ecosystems, serving a variety of functions (e.g. herbivores, parasitoids, predators, and pollinators) (Sharkey 2007; Blaimer et al. 2023). The remarkable diversity of species and the apparent changes in lifestyles have prompted interest in the evolution of Hymenoptera. The current phylogenetic analyses based on various datasets have largely established the phylogenetic framework of Hymenoptera and identified several key innovative events in their evolution (Branstetter et al. 2017; Peters et al. 2017; Tang et al. 2019; Blaimer et al. 2023). In brief, the origin of the Hymenoptera is estimated to be approximately 281 million years ago (Mya) in the Permian period (Peters et al. 2017). It is likely that the ancestral hymenopterans are plant-feeders, and this lifestyle is still retained in the majority of sawflies, which represent the early branches of the Hymenoptera tree (Peters et al. 2017; Blaimer et al. 2023). The emergence of parasitoidism occurred in the last common ancestor of Orussoidea and wasp-waisted wasps (also known as Apocrita) (Peters et al. 2017; Blaimer et al. 2023; Ye et al. 2024). This transition from phytophagy to parasitoidism is regarded as a key innovation in the evolution of Hymenoptera and may have been a significant driver of species diversification, as approximately 70% of all described hymenopterans are parasitoids (Peters et al. 2017; Blaimer et al. 2023; Polaszek and Vilhemsen 2023; Ye et al. 2024). In Apocrita, species have evolved a highly constricted “waist” between the first and second abdominal segments, and this wasp-waist allows for flexibility in female ovipositors, which may be an important adaptation for parasitoid lifestyles (Vilhelmsen et al. 2010; Blaimer et al. 2023). Another morphological innovation is the acquisition of the venomous stinger (the modified female ovipositor from an egg-laying apparatus to a stinging apparatus) in Aculeata (Peters et al. 2017; Blaimer et al. 2023). And some stinging species in the Aculeata (e.g. ants and certain groups of bees and wasps) have also evolved into eusociality independently (Peters et al. 2017). Furthermore, it is important to note that following the evolution of the basal hymenopteran diet from phytophagy to parasitoidism (equivalent to carnivory), certain gall wasps, fig wasps, and pollen-collecting bees have independently evolved again to phytophagy (i.e. secondary phytophagy) (Blaimer et al. 2023). A recent phylogenetic study has indicated that the evolution of secondary phytophagy had a significant influence on the diversification rate in Hymenoptera (Blaimer et al. 2023). Overall, the evolution of Hymenoptera has been accompanied by several key innovations and evolutionary changes of lifestyles; however, the genetic mechanisms underlying these evolutionary processes remain largely unknown.

The sequencing of numerous hymenopteran genomes has opened new avenues for a deeper understanding of Hymenoptera evolution, yet a comprehensive, large-scale comparative genomic analysis of Hymenoptera is lacking. Previous studies have focused on narrow taxonomic groups (e.g. ants and bees) (Fouks et al. 2021; Lin et al. 2021; McKenzie et al. 2021) or specific traits (e.g. eusociality) (Woodard et al. 2011; Kapheim et al. 2015; Jones et al. 2023), leaving a critical gap in our knowledge of how genomic changes have driven the order's macroevolutionary success. To fill this gap, we performed a large-scale comparative genomics study containing 131 species, which represent major lineages of Hymenoptera. Using the genomic data, we systematically investigated: (i) the general patterns of genome evolution in terms of gene content and evolutionary rate throughout hymenopteran history; (ii) genomic changes that occurred at the evolutionary branches associated with key innovations; (iii) differences in genome evolution between two major clades in the Hymenoptera phylogeny, the Parasitoida and Aculeata; and (iv) genomic features related to three independently evolutionary events of secondary phytophagy. Our findings not only offer a comprehensive picture of the genome evolution of hymenopteran insects but also highlight several genomic changes potentially related to evolutionary innovations, shedding light on the genomic basis of adaptation and diversification within this remarkable order.

## Results and Discussion

### Hymenoptera Phylogenomics

**Fig. 1.**
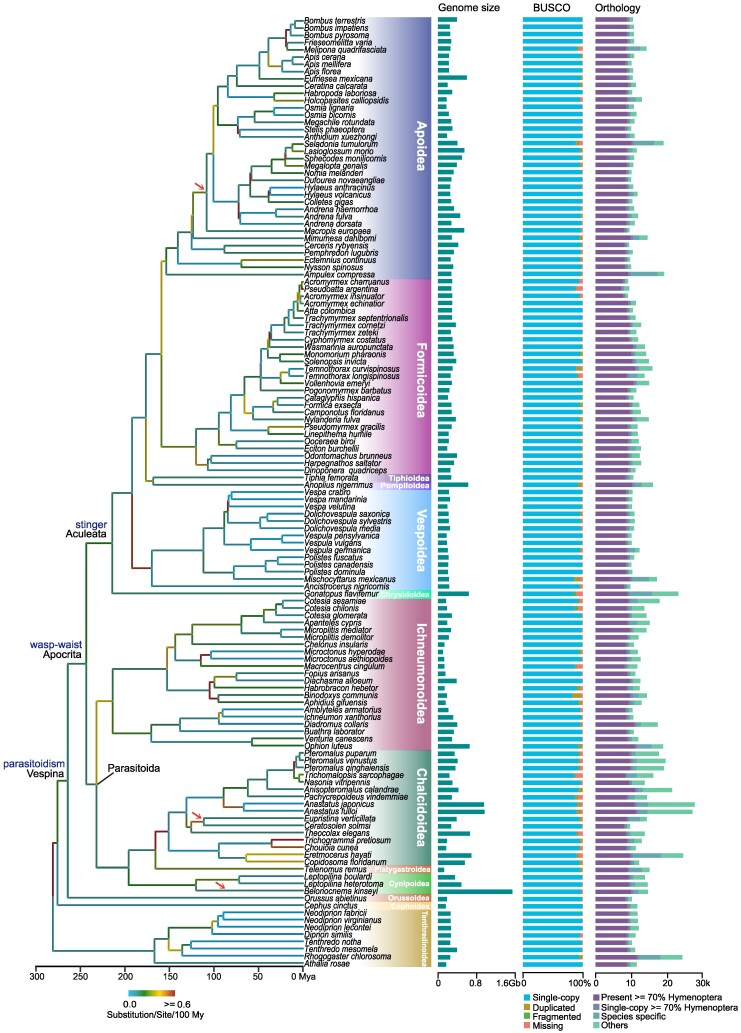
Phylogenetic analysis and genomic comparisons of Hymenoptera. The maximum-likelihood phylogenetic tree on the left was constructed with 1,000 ultrafast bootstraps under a JTT + F + R10 model using protein sequences from 1,002 single-copy genes. Almost all the nodes received 100% bootstrap support, except for nine. The branch shading (as indicated by the scale bar) corresponds to the rate of substitutions per site per 100 My along that particular branch. The divergence times among species were estimated using 13 calibration points. Several key innovations, such as parasitoidism, wasp-waist, and stinger, are marked on the phylogeny, and three independent transitions to secondary phytophagy are indicated by red arrows. On the right side, the figure shows (i) genome assembly sizes of hymenopteran species; (ii) BUSCO profiles for these genomes; and (iii) orthology profiles. In the orthology analysis, the protein-coding genes of each species were subdivided to represent different types of orthology clusters.

To explore the evolutionary history of Hymenoptera, we obtained high-quality genomes for 131 hymenopteran insects from 13 superfamilies (29 families), covering many representative lineages with unique lifestyles such as sawflies, wasps, ants, and bees (Supplementary table S1, Supplementary Material online and Supplementary fig. S1, Supplementary Material online). These genomes were all publicly available, and 78 of them were sequenced using long-read sequencing technologies, showing the overall high contiguity (mean Contig N50 4.23 Mb). Furthermore, BUSCO (Benchmarking Universal Single-Copy Orthologs) assessments indicated that these genomes have a high level of gene completeness (highest 99.80%, lowest 85.10%, mean 97.26%, SD = 0.03) (Supplementary table S1, Supplementary Material online). In order to minimize the impact of potential gene annotation issues on subsequent analysis, we then employed an alignment-based approach to identify potential broken and chimeric genes (Mantica et al. 2024), correcting about 3.6% of total genes (Supplementary table S2, Supplementary Material online). After gene corrections, orthology inference produced 33,045 orthogroups (OGs, also referred to as gene families), with 25.4% of them present in over 70% of hymenopterans (Fig. 1, Supplementary fig. S2, Supplementary Material online, and Supplementary table S3, Supplementary Material online). Of the total number of genes analyzed, 78,789 (4.77%) were identified as singletons, meaning that no homolog was predicted in any genome.

We next reconstructed the phylogeny of hymenopterans based on protein sequences from 1,002 single-copy genes under a best-fitting JTT + F + R10 model (Fig. 1). Only nine (6.98%) nodes in this tree failed to receive 100% bootstrap support (Supplementary fig. S3, Supplementary Material online). Our species tree was mostly consistent with previous hymenopteran phylogenies using transcriptomic, mitochondrial data, and BUSCO proteins (Peters et al. 2017; Tang et al. 2019; Guinet et al. 2023). We showed that: (i) phytophagous sawflies from Tenthredinoidea and Cephoidea are placed in the early branches of Hymenoptera; (ii) parasitoid Orussoidea is a sister to all wasp-waisted wasps, also known as Apocrita; and (iii) in Apocrita, parasitoid wasps from several superfamilies such as Ichneumonoidea, Chalcidoidea, Cynipoidea, and Platygastroidea form one clade (i.e. Parasitoida), and stinging species of Aculeata form its sister clade (Fig. 1). The phylogenetic relationship within Apocrita, where parasitoid wasps form a clade sister to the stinging Aculeata, differs from the results of studies using ultra-conserved elements (Branstetter et al. 2017; Blaimer et al. 2023). These studies support that Ichneumonoidea is a sister to the clade containing all other species of Apocrita. There may be several reasons for this conflict, such as the type of data used and the samples analyzed. Our study represents only a small fraction of the total documented hymenopteran species. Further studies should expand the sampling efforts to establish a more robust tree for Hymenoptera. In our subsequent analyses, we used the phylogenetic tree constructed from the genomic data, and the divergence times were estimated using 13 calibration points based on a previous study (Peters et al. 2017) (Fig. 1 and Supplementary fig. S3, Supplementary Material online).

### Genomic Changes Throughout Hymenoptera History

We next investigated the changes in three different genomic features of protein-coding genes, including gene family evolution, protein domain rearrangement, and the evolutionary rate of protein-coding genes, to explore the overall dynamics of Hymenoptera genome evolution. First, we inferred the history of gene family evolution and identified 63,907 gene family expansions and 106,004 contractions (Supplementary figs S4 and S5, Supplementary Material online). This pattern suggests a generally reductive mode of gene family evolution in Hymenoptera, which is also supported by a small-scale comparative genomic study of this order (Oeyen et al. 2020). The pincer wasp *Gonatopus flavifemur* (Chrysidoidea, Dryinidae) is notable among hymenopterans for having the largest number of gene family expansion (736) and contraction (2,564) events (Supplementary fig. S4, Supplementary Material online). We also estimated rates for gene family size evolution and found the average global rate of gene gain and loss of gene families in Hymenoptera was estimated to be 58.29 gene gain and loss per million years (My). The rates of most hymenopteran branches are relatively comparable, but we also noticed several notable accelerations of the gene gain and loss rates in different lineages. For example, the clade comprising *Nasonia*, *Trichomalopsis*, and *Pteromalus* in Pteromalidae exhibited an average gene gain and loss rate of 605.74 per My. Furthermore, a noticeable acceleration in gene gain and loss rates can be observed in the branches of leafcutter ants (Formicidae), as previously noted in studies (Wissler et al. 2013; Thomas et al. 2020) (Supplementary figs S6 and S7, Supplementary Material online and Supplementary table S4, Supplementary Material online).

We identified 230 gene families that exhibited significant expansions or contractions during Hymenoptera evolution, and Gene Ontology (GO) enrichment analysis showed that they were enriched for the steroid metabolic process, the maltose metabolic process, and sensory perception of smell (FDR-adjusted *P* < 0.05; Supplementary tables S5 and S6, Supplementary Material online and Supplementary fig. S8, Supplementary Material online). Among these, several rapidly evolved digestion-related gene families, such as Maltase, Esterase, and Trypsin, may be associated with adaptation to different diets in Hymenoptera (Supplementary table S5, Supplementary Material online). The rapid evolution of the Fatty acyl-CoA reductase family involved in the biosynthesis of insect pheromones may be driving the evolution of pheromone diversity (Teerawanichpan et al. 2010; Carot-Sans et al. 2015; Dou et al. 2020). The significant expansions and contractions of Odorant receptor families may be related to the rapid adaptations to detect the different odors from the living environments (such as pheromones and odors related to host plants or host insects) of diverse hymenopterans. We also found that the size of the cuticular protein family changed rapidly during the Hymenoptera evolution (Supplementary fig. S9, Supplementary Material online), which might be associated with the evolutionary diversity of the cuticular exoskeleton and morphology (Xiong et al. 2017; Pan et al. 2018). Detailed analysis further indicated that the size variation of the cuticular protein family mainly occurred in the CPR-RR-1, CRP-RR-2, and CPAP1 subfamilies (Supplementary table S7, Supplementary Material online and Supplementary fig. S9, Supplementary Material online).

We also investigated the evolution of protein domain families based on the Pfam annotations. Similarly, we identified 105 Pfam domain families with significantly accelerated rates, including OS-D (Insect pheromone-binding family, A10/OS-D, PF03392.12), FA_desaturase (Fatty acid desaturase, PF00487.23), Chitin_bind_4 (Insect cuticle protein, PF00379.22), 7tm_2 (7 transmembrane receptor, PF00002.23), and Lipase (Lipase, PF00151.18), consistent with the significantly changed gene families (Supplementary table S8, Supplementary Material online). Furthermore, we reconstructed the rearrangements of protein domains in Hymenoptera evolution (see Materials and Methods). In total, 33,943 domain arrangement changes were observed, and 41.8% of them were formed by a fusion of two ancestral domains (Supplementary table S9, Supplementary Material online). We noticed the higher rates of domain rearrangements in several lineages, such as *G. flavifemur*, *Temnothorax longispinosus*, and *Diadromus collaris*, which may contribute to the evolutionary innovations of these species (Supplementary table S9, Supplementary Material online and Supplementary fig. S10, Supplementary Material online).

**Fig. 2.**
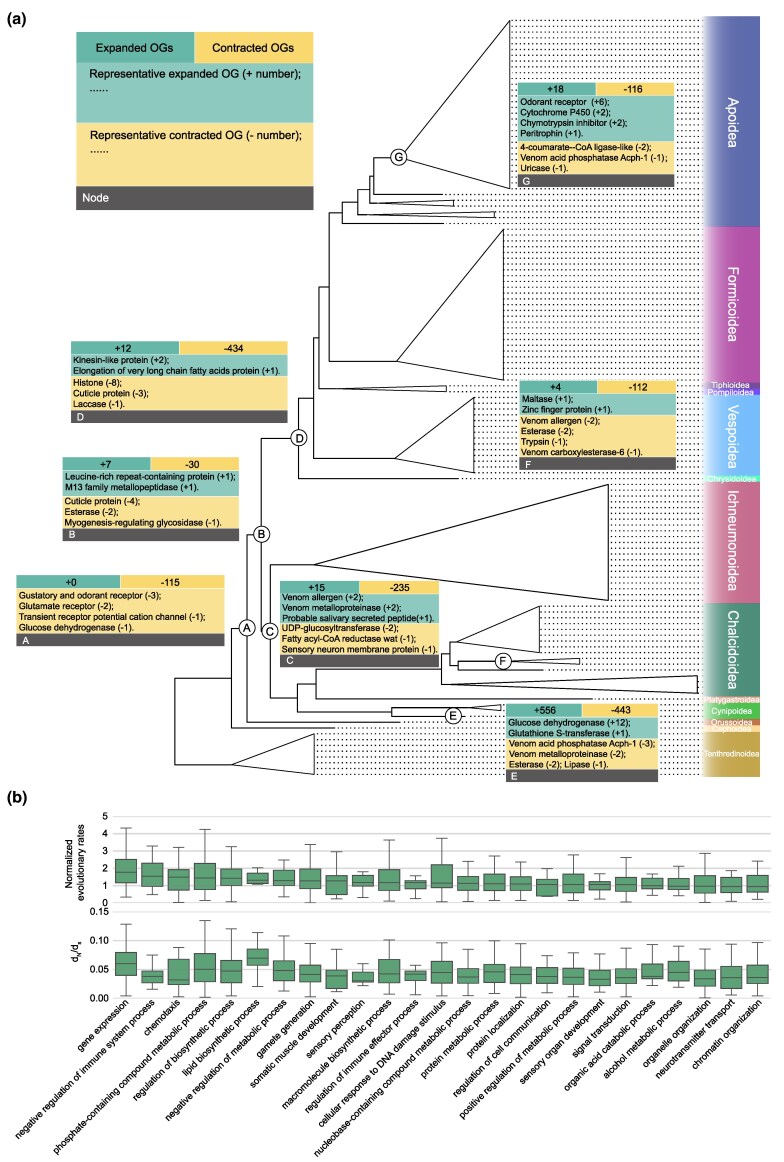
Gene family evolution and rapidly evolving genes in Hymenoptera. a) Gene family expansion and contraction at the nodes of key innovations in Hymenoptera evolution. In each box, the first row shows the number of gene families that expanded or contracted, respectively. Below are the corresponding representative gene families, and the size changes of each gene family are also indicated. b) Rapidly evolving OGs and functional categories in terms of evolutionary rate and selection constraint (*d*_N_/*d*_S_). Functional categories (GO Biological Process terms) are sorted by normalized evolutionary rates, and the top 25 GO terms are shown. The normalized evolutionary rate of each OG is measured as the mean of the amino acid substitution rates of each OG normalized by the genome-wide amino acid substitution rates. The *d*_N_/*d*_s_ of each OG refers to the ratio of the nonsynonymous substitution rate to the synonymous substitution rate. OGs are further grouped according to their functional similarity.

We next explored the changes in the evolutionary rate of the protein-coding genes in Hymenoptera. We first estimated the rates of amino acid substitutions across the Hymenoptera phylogeny to reveal the overall pattern of protein sequence evolution (Fig. 1). Our analysis estimated an average amino acid substitution rate of 0.0025 substitutions per site per My with a standard deviation of 0.0055 (Supplementary table S10, Supplementary Material online). We found that the overall accelerated protein evolution events were dispersed across the multiple lineages of Hymenoptera, such as some clades in Anthophila (Apoidea) and Braconidae (Ichneumonoidea). These evolutionary rate shifts may be related to phenotypic transitions. For example, a comparative genomic study has suggested that the accelerated evolution of a number of proteins is linked to the independent miniaturization across parasitoid wasps (Xu et al. 2021). Then, we examined the evolutionary rate of each orthologous protein-coding gene by measuring both the normalized amino acid substitution rates and the selective constraint (ratio of nonsynonymous to synonymous substitutions, *d*_N_/*d*_S_). Functional analysis showed that genes associated with gene expression evolved most rapidly, followed by genes related to immunity, metabolism, and sensory perception (Fig. 2b). The rapid evolution of genes in these functional categories may play a role in phenotypic innovation and adaptive evolution in Hymenoptera. In contrast, genes with slower evolutionary rates were mainly enriched in some housekeeping processes, such as cellular component organization and cell development (Supplementary fig. S11, Supplementary Material online). In addition, we also conducted whole genome alignment analysis (Cactus alignment) to investigate the evolutionary constraints and acceleration at the whole genome level (see Materials and Methods). We chose the bumblebee *Bombus terrestris* (Apidae) as a reference because the availability of ATAC-Seq data is helpful to infer potential regulatory elements (Zhao et al. 2020). In the 27-way alignment, 59.5% (233.69/392.96 Mb) of the *B. terrestris* genome was aligned to at least one species, and 21.5% and 8.5% of the genome were identified as constrained and accelerated regions, respectively, by calculating the phyloP score (Supplementary fig. S12a, Supplementary Material online). We then identified the top 5% most accelerated and most conserved genes, measured by the average phyloP score of the coding sequence of each gene (Supplementary table S11, Supplementary Material online). GO enrichment analyses indicated that the most constrained genes were enriched in structure morphogenesis and development, while the most accelerated genes were involved in sensory perception and steroid metabolic processes, consistent with our previous findings in Fig. 2b (Supplementary figs S12b and S12c, Supplementary Material online and Supplementary tables S12 and S13, Supplementary Material online). We also identified 481,924 highly conserved elements using phastCons, and 27,602 (5.7%) of them were conserved across all species in our 27-way alignment (Supplementary fig. S13a, Supplementary Material online). Among them, 151 noncoding regions were overlapped with the accessible chromatin regions supported by ATAC-Seq in the reference species *B. terrestris*, suggesting their potential functions in gene regulation. We further found that the genes located near these conserved noncoding elements were functionally related to nervous system development and cell morphogenesis (FDR-adjusted *P* < 0.05; Supplementary fig. S13b, Supplementary Material online and Supplementary table S14, Supplementary Material online).

### Genomic Characteristics Accompanying Key Innovations in Hymenoptera Evolution

We next examined several key nodes in the evolutionary history of Hymenoptera, investigating the genomic changes that occurred at these nodes with the aim of correlating the association of genomic changes and phenotypic innovations. Here, we focused on four key evolutionary positions: the origin of parasitoidism (node A in Fig. 2a), the emergence of the wasp-waist (node B), the stem of Parasitoida that is prior to the radiation of the majority of parasitoid wasps (node C), and the emergence of the stinger (node D). The genomic features related to secondary phytophagy in several independent lineages will be discussed in a subsequent section. We did not investigate the genome evolution of eusocial hymenopterans due to the significant contributions of some recent works in this area (Woodard et al. 2011; Kapheim et al. 2015; Jones et al. 2023; Lacy and Kronauer 2023; Wyatt et al. 2023; Mikhailova et al. 2024).

We first focused on the last common ancestor of the parasitoid wood wasp *Orussus abietinus* (Orussidae) and other wasp-waisted species (Apocrita), which represents the position of parasitoidism origin in the Hymenoptera tree. Our analysis identified 115 contracted and no expanded gene families at this node (Fig. 2a and Supplementary table S15, Supplementary Material online). Furthermore, we also found 137 missing gene families (i.e. gene families present in over 70% of outgroup species and lost in the test group) and only five novel gene families (also known as novel core genes, which are retained in over 70% of species in the test group) (Supplementary tables S16 and S17, Supplementary Material online). This pattern of gene family evolution suggests that the reductive evolution of gene families may have played an important role in the origin of parasitoidism. It is important to note that while we implemented various procedures to minimize false positives in identifying missing gene families, we recognize that several factors may still influence these identifications, such as the quality of genomic annotation and the accelerated sequence evolution observed in certain lineages. Future work is essential to conduct more in-depth investigations into gene loss across Hymenoptera. GO enrichment analysis showed that these contracted and missing families were significantly enriched for functions associated with detection of stimulus, cholesterol transport and metabolism, lipid metabolism, and xenobiotic metabolic processes (FDR-adjusted *P* < 0.05; Supplementary tables S18 and S19, Supplementary Material online). The changes in these gene families in the ancestral parasitoid wasp could be associated with the shift in lifestyle and diet from a phytophagous sawfly to an insectivorous parasitoid. For instance, the genes related to the detection of stimulus (e.g. Gustatory and odorant receptors, Glutamate receptors, and Transient receptor potential channels) may contribute to early parasitoid wasps locating potential hosts in novel environments (i.e. in dead wood for most parasitoids in Orussidae; Oeyen et al. 2020). The contracted gene families with cholesterol-transporting and metabolizing functions may be associated with alterations in the nutritional composition of the foods consumed after the acquisition of parasitoidism. In addition, we identified 96 genes that show evidence of positive selection in the parasitoid ancestor (aBSREL, FDR-adjusted *P* < 0.05; Supplementary table S20, Supplementary Material online). These genes were associated with many developmental processes, including muscle tissue development, eye morphogenesis, respiratory system development, and larval salivary gland morphogenesis (Supplementary table S21, Supplementary Material online). These genomic changes may be related to the dramatic changes in morphological characteristics that are considered adaptations to a parasitoid lifestyle, such as simplified larval morphology lacking eyes and legs in Orussidae (Vilhelmsen 2003; Oeyen et al. 2020).

In the last common ancestor of wasp-waisted species (Apocrita), we also identified more contracted gene families (30) than expanded gene families (7) (Fig. 2a and Supplementary table S22, Supplementary Material online). The contraction of Cuticle protein genes that have roles in cuticle development at the Apocrita stem may contribute to the emergence of wasp-waist. We also discovered 119 positively selected genes with diverse functions such as endocytosis and regulation of enzyme activity (aBSREL, FDR-adjusted *P* < 0.05; Supplementary table S23, Supplementary Material online). At the stem of stingers, the Aculeata, 434 contracted gene families were found, but only 12 expanded gene families were found (Supplementary table S24, Supplementary Material online). The expanded genes included the elongation of very long chain fatty acid protein genes that are involved in female pheromone biosynthesis (Chertemps et al. 2007; Chen et al. 2023b). The contracted genes with functions related to cuticle development (e.g. Cuticle protein genes and Laccase genes) were also identified. Moreover, our analysis revealed 175 positively selected genes at the stem of the Aculeata (Supplementary table S25, Supplementary Material online). These included the genes that regulate the highly conserved pathways for development, such as Wnt signaling pathway (Rim et al. 2022) (e.g. *FZD2*, *LRP6*, and *MLLT3*) and Notch signaling pathway (Zhou et al. 2022) (e.g. *Ift172*, *NCSTN*, and *POFUT1*). Changes in these genes may have contributed to the unique morphological features of stinging hymenopterans.

The Parasitoida includes a large number of parasitoid wasp species, and the stem of the Parasitoida clade showed 235 contracted and 15 expanded gene families (Fig. 2a and Supplementary table S26, Supplementary Material online). We found the expansion of Venom allergen genes and Venom metalloproteinase genes, which may contribute to venom functions to enhance the probability of parasitic success. Additionally, we inferred that the expanded Salivary secreted peptide family may also play a role in feeding on hosts and regulation of host immunity, based on current knowledge of the functions of the salivary proteins in parasitoid wasps (Richards 2012; Shi et al. 2022; Pang et al. 2023). This result suggests that the expansion of gene families involved in parasitoid–host interaction occurred prior to the radiation of parasitoid wasps. Taken together, our findings provide a number of candidate genes that may be linked to the evolution of key innovations in Hymenoptera.

### Divergent Trajectories of Genomic Change Between Parasitoida and Aculeata

The Parasitoida and Aculeata clades diverged from the ancestral wasp-waisted wasp at around 243 Mya, representing two major groups of hymenopterans with clear differences in lifestyle, morphology, and diversity (Peters et al. 2017) (Fig. 1). The Parasitoida clade includes primarily parasitoid wasps that lack a stinger, and the Aculeata clade comprises stinging species with diverse life habits, including stinging parasitoids, social wasps, ants, and bees (Peters et al. 2017; Polaszek and Vilhemsen 2023). We next sought to explore and compare the genomic changes within these two clades, which may provide insights into the independent evolution of two major groups of hymenopterans.

**Fig. 3.**
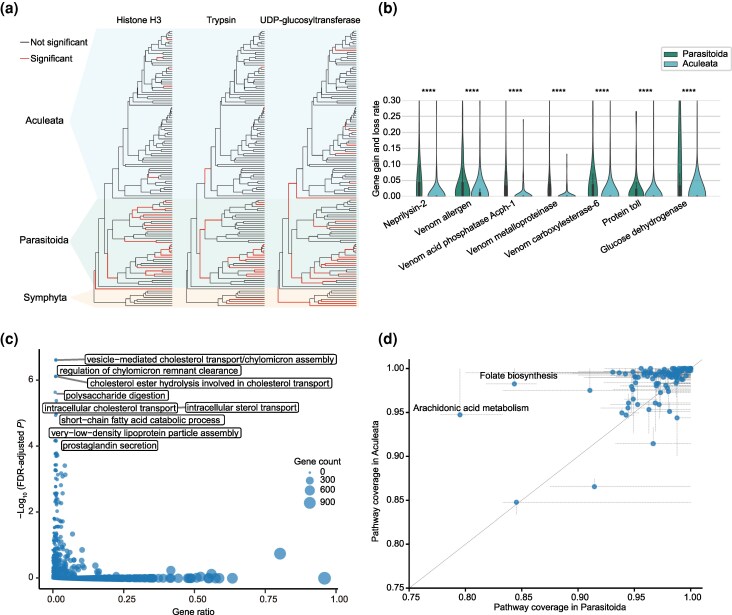
Different evolutionary genome dynamics between Parasitoida and Aculeata. a) The distribution of the rapidly evolving events for gene families on the branches of the Parasitoida and Aculeata clades. Three gene families, Histone H3, Trypsin, and UDP-glucosyltransferase, are shown here, which have a significantly higher occurrence rate of rapid evolutionary events in Parasitoida than in Aculeata (FDR-adjusted *P* < 0.05, Chi-square test, odds ratio > 1). b) Gene families that have a higher rate of gene gain and loss in Parasitoida than in Aculeata. The gene gain and loss rate is measured as the sum of gene gains and losses per million years. Asterisks indicate significant shifts between Parasitoida and Aculeata (One-tailed Mann–Whitney *U* test; *≤0.05; **≤ 0.01; *** ≤ 0.001; **** ≤ 0.0001). c) GO enrichment analysis of the gene families with a faster rate of gene gain and loss in Parasitoida than in Aculeata. The box shows the GO terms for the 10 biological processes with the lowest adjusted *P* value (FDR-adjusted *P* < 0.05, Benjamini–Hochberg multitest correction). The size of the points represents the number of genes in the corresponding GO. d) KEGG pathway coverage comparison between Parasitoida and Aculeata. The expanded lines from the point represent the first and third quartiles of pathway coverage in Parasitoida and Aculeata, respectively.

Depending on the results of gene family evolution, we determined the distribution of the rapidly evolving events for each gene family on the branches in the Parasitoida and Aculeata clades. This thus allowed us to explore whether there are gene families with a clear distributional preference for rapid evolutionary events in the two clades. A total of 91 gene families were identified that exhibited rapid evolutionary events, with a significantly higher prevalence observed in the Parasitoida compared to the Aculeata (FDR-adjusted *P* < 0.05, Chi-square test, odds ratio > 1; Fig. 3a, Supplementary fig. S14, Supplementary Material online, and Supplementary table S27, Supplementary Material online). These included a number of Histone genes (i.e. H2A, H2B, H3, and H4) that are involved in the structure of chromatin (Peterson and Laniel 2004; Martire and Banaszynski 2020), suggesting the potential distinct chromatin structures in parasitoid wasps. The enrichment of rapidly evolving Trypsin, Glucose dehydrogenase, and UDP-glucosyltransferase families in the Parasitoida may be associated with the evolutionary adaptations of digestion and detoxification metabolism, enabling the parasitoid wasps to adapt to diverse arthropod hosts. The rapid evolution of the Peptidoglycan recognition protein family in the Parasitoida may contribute to the antimicrobial defenses of parasitoid wasps (Kurata 2014; Mei et al. 2022). Furthermore, we observed significant changes in the SET and MYND domain-containing protein family, an epigenetic regulator (Xiao et al. 2018), in the Parasitoida, which suggests that epigenetic regulation may play an important role in the evolution of parasitoid wasps. By contrast, only 12 gene families were identified as enriched rapidly evolving families in the Aculeata, including the THAP domain-containing protein, RCC1 and BTB domain-containing protein, Odorant receptor, and Gustatory receptor (FDR-adjusted *P* < 0.05, Chi-square test, odds ratio > 1; Supplementary table S28 and Supplementary fig S15, Supplementary Material online). In addition, we estimated and compared the rates of gene gain and loss for each gene family between the Parasitoida and Aculeata clades to determine the gene families with significantly different gene gain and loss rates within the two clades. This analysis may capture additional gene families that have undergone significant evolutionary changes between the Parasitoida and Aculeata, which may have been overlooked in previous analyses due to the limited number of evolutionary branches where such changes occurred. In this analysis, we identified 1,604 gene families with significantly higher rates of gene gain and loss in the Parasitoida compared to the Aculeata (FDR-adjusted *P* < 0.05, One-tailed Mann–Whitney *U* test; Fig. 3b and Supplementary table S29, Supplementary Material online). GO analysis of the gene families with high turnover rates in the Parasitoida showed enrichment for functional categories such as polysaccharide digestion, intracellular sterol transport, and prostaglandin secretion (FDR-adjusted *P* < 0.05; Fig. 3c and Supplementary table S30, Supplementary Material online). Additionally, we noticed some genes related to venom functions were included in this gene set, suggesting venom may play a key role in the evolution and diversity of parasitoid wasps in Parasitoida (Fig. 3b; discussed in detail in the next section). Similarly, we found a small number of gene families (26) with faster gene gain and loss rates in the Aculeata compared to the Parasitoida (FDR-adjusted *P* < 0.05, One-tailed Mann–Whitney *U* test; Supplementary table S31, Supplementary Material online).

We next compared the selective constraint (*d*_N_/*d*_S_) of single-copy OGs between the Parasitoida and Aculeata. The result showed none of the OGs had significantly different *d*_N_/*d*_S_ between the two clades (Two-tailed Mann–Whitney *U* test; Supplementary table S32, Supplementary Material online). We hypothesized that this result may be attributed to the presence of significant variability in the *d*_N_/*d*_S_ ratio of the branches within the two clades. In order to investigate the degree of variation in *d*_N_/*d*_S_ between the Parasitoida and Aculeata, we calculated the coefficient of variation (CoV) of the *d*_N_/*d*_S_ in the two clades (Supplementary table S33, Supplementary Material online). Our findings revealed that the *d*_N_/*d*_S_ variation of the Parasitoida was significantly larger than that of the Aculeata (*P* = 7.92e−84, One-tailed Mann–Whitney *U* test; Supplementary fig. S16, Supplementary Material online). And 73.8% of the analyzed OG exhibited a greater CoV of the Parasitoida than that observed in the Aculeata (Supplementary table S33, Supplementary Material online). This result suggests that the Parasitoida may be subject to more variable selection pressures compared to the Aculeata. This is likely to be related to the diverse lifestyles and hosts of the parasitoid wasps in Parasitoida. We also identified 13 OGs with obviously higher CoV of the *d*_N_/*d*_S_ in the Parasitoida (i.e. the OGs with the top 10% CoV in Parasitoida and the bottom 10% CoV in Aculeata), including exocytosis-related STXBP5, calcium-binding protein Calmodulin, and cytoskeleton organization-related DIAPH, suggesting that they may have evolved under different selective pressures and may play key roles in Parasitoida evolution (Supplementary table S34, Supplementary Material online).

We further explored the variation in metabolic pathways between the Parasitoida and Aculeata, which may reflect differences in the life history of these two groups. To compare the pathway repertoire variation between the two clades, we first performed EC (Enzyme Commission number) annotations for all 131 hymenopterans (Supplementary table S35, Supplementary Material online) and constructed ancestral KEGG (Kyoto Encyclopedia of Genes and Genomes) pathways based on eight phytophagous sawflies (Tenthredinoidea) located on the early branches of the Hymenoptera tree (see Materials and Methods). Then, we mapped the present/absent information of each EC of the species from the Parasitoida and Aculeata to the ancestral pathways. This allows us to study the pathway-level changes of Parasitoida and Aculeata after their divergence. We examined the pathway coverage (PC, i.e. the fraction of ECs in an ancestral pathway that were annotated in a species) of each pathway in each species (Supplementary table S36, Supplementary Material online). Overall, our results showed significantly lower PC for Parasitoida compared to Aculeata (*P* = 2.68e−46, One-tailed Mann–Whitney *U* test; Supplementary fig S17, Supplementary Material online), suggesting that the specialized parasitoidism may have simplified the pathways of parasitoid wasps, and similar phenomena have also been demonstrated in other parasitic species, such as parasitic worms (International Helminth Genomes Consortium 2019), dicyemids (Lu et al. 2019), protists (Ralton et al. 2021), and plants (Chen et al. 2023a). Our detailed analysis further revealed that 37 pathways from 14 KEGG superpathways exhibited significantly lower PC in Parasitoida when compared to Aculeata, which are involved in lipid metabolism, metabolism of cofactors and vitamins, carbohydrate metabolism, etc. (FDR-adjusted *P* < 0.05, One-tailed Mann–Whitney *U* test; Fig. 3d, Supplementary fig. S18, Supplementary Material online, and Supplementary table S37, Supplementary Material online). Conversely, five pathways showed lower PC for Aculeata, and these metabolic processes included purine metabolism, pentose phosphate pathway, and glyoxylate and dicarboxylate metabolism (FDR-adjusted *P* < 0.05, One-tailed Mann–Whitney *U* test; Fig. 3d, Supplementary fig S18, Supplementary Material online, and Supplementary table S38, Supplementary Material online). Among these PC variations, we inferred the lower PC of arachidonic acid metabolism (ko00590) and folate biosynthesis (ko00790) in Parasitoida caused by the pervasive absence of carbonyl reductase (K00079, EC. 1.1.1.184/189/197). In addition, we found that the loss of the same enzyme may have different effects on these two pathways. Specifically, the absence of carbonyl reductase in the arachidonic acid metabolic pathway has disrupted the pathway from prostaglandin E2 to prostaglandin F2alpha. The widespread absence of this pathway in parasitoid wasps suggests that they may have other ways of acquiring prostaglandin F2alpha, probably from their diet. However, the absence of carbonyl reductase in the folate biosynthetic pathway did not result in pathway disruption due to the redundancy in this pathway. This may represent a streamlined evolution of the pathway in parasitoid wasps. We next compared the CoV of the PC between the two clades and found six superpathways exhibited significantly higher variation in Parasitoida, also covering the pathways we discussed above (FDR-adjusted *P* < 0.05, One-tailed Mann–Whitney *U* test; Supplementary fig S19, Supplementary Material online). This finding suggests that these pathways are more variable in parasitoid wasp species, and it is likely that these variations occurred several times independently in Parasitoida evolution. This may suggest that the rapid changes in these pathways are related to the adaptation of parasitoid wasps to diverse parasitoid lifestyles, such as adaptation to different hosts and different parasitic strategies.

### Massively Duplicated Genes and Gene Losses in Parasitoida

The preceding section of this study has demonstrated that parasitoid wasps exhibit high rates of gene gain and loss, which likely results in significant changes in the number of members of certain gene families. Here we first explored the gene families with significantly larger gene numbers in Parasitoida than those in other taxa, which were likely to be caused by the massive gene duplications that occurred in Parasitoida. In total, we identified 95 gene families with a significantly larger number of genes in Parasitoida species compared to other hymenopterans (FDR-adjusted *P* < 0.05, One-tailed Mann–Whitney *U* test; Supplementary table S39, Supplementary Material online and Supplementary fig. S20, Supplementary Material online). Functional annotation of these families was diverse, but we observed they were frequently related to chemoreception (such as Odorant receptor, Gustatory receptor, and General odorant-binding protein), detoxification (such as Multidrug resistance-associated protein and UDP-glycosyltransferase), and protein degradation (such as Proteases and Protease inhibitors). Among these gene families, we also found 41 families with no annotation information, designated as Putative uncharacterized proteins. In contrast, we only found nine enlarged gene families for Aculeata, with three families belonging to the Odorant receptor (Supplementary fig. S21, Supplementary Material online and Supplementary table S40, Supplementary Material online). The findings suggest that gene duplication events were more prevalent in Parasitoida, and the identified gene families that exhibited massive expansions may have played roles in the adaptive evolution of parasitoid wasps and promoted the diversity of these species.

**Fig. 4.**
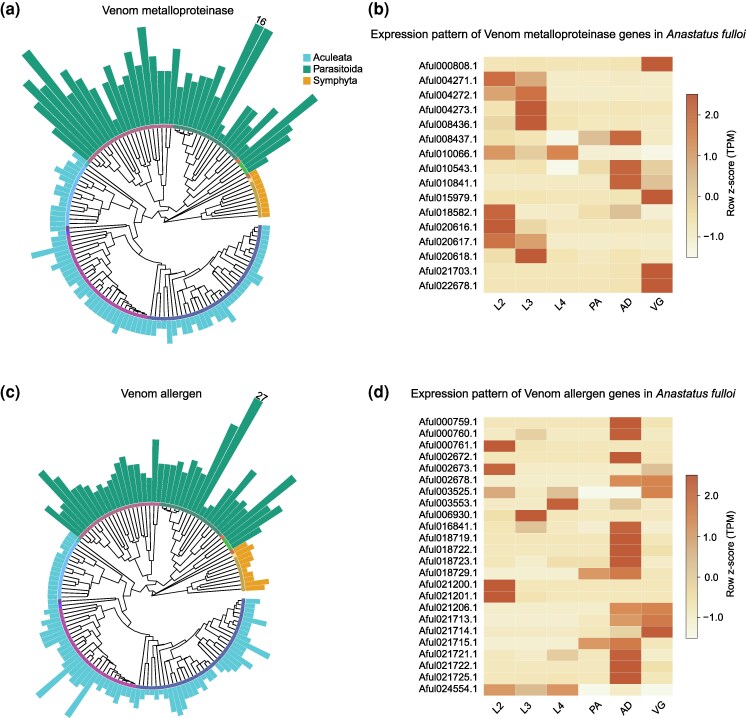
Expansions of Venom allergen and Venom metalloprotease gene families in Parasitoida. a) The size distribution of the Venom metalloprotease gene families in Hymenoptera. The number on the bar indicates the maximum gene copy count across the species for that family. b) Expression pattern of the Venom metalloprotease genes in *A. fulloi* at five developmental stages and the venom gland. Expression is measured using *z*-score values of TPM by row. L2, second instar larva; L3, third instar larva; L4, fourth instar larva; PA, pupal stage; AD, female adult; VG, venom gland. Only genes with a TPM greater than 0 in at least one period are shown. c) The size distribution of the Venom allergen gene families in Hymenoptera. d) Expression patterns of the Venom allergen genes in *A. fulloi* at five developmental stages and the venom gland.

Among the gene families identified with large-scale gene duplications in Parasitoida, we found several families whose members are often known as venom components, the important weapons of parasitoid wasps to overcome host immune systems or manipulate host development (Asgari and Rivers 2011; Moreau and Asgari 2015). For instance, our analysis indicated that the Venom metalloproteinase family has undergone an extensive expansion, specifically in the Parasitoida clades, which may have contributed to the recruitment of the family member as a venom component in parasitoid wasps through neofunctionalization (Fig. 4a and Supplementary fig. S22, Supplementary Material online). Indeed, Venom metalloproteinase has been reported as a venom protein in at least 19 parasitoid wasps from five families of three superfamilies in Parasitoida (Parkinson et al. 2002; Price et al. 2009; Vincent et al. 2010; Werren et al. 2010; Dorémus et al. 2013; Formesyn et al. 2013; Goecks et al. 2013; Burke and Strand 2014; Teng et al. 2017; Zhao et al. 2017; Lin et al. 2019; Alvarado et al. 2020; Dennis et al. 2020; Yang et al. 2020; Gatti et al. 2021; Ye et al. 2023; Yu et al. 2023). And some studies have demonstrated that these venom proteins play distinct roles (such as regulating host development [Price et al. 2009] and inhibiting host immunity [Lin et al. 2018]) in different parasitoid–host systems, suggesting the duplications of this family in parasitoid wasps may also be related to the diversity of venom functions. Additionally, the duplication of Venom metalloproteinase genes may also help diversify nonvenomous functions. In *Anastatus fulloi* (Chalcidoidea, Eupelmidae), our gene expression profiling showed diverse expression patterns of Venom metalloproteinase genes, suggesting that they have functions other than venom functions (Fig. 4b). For example, nine of them exhibited specifically higher expression in the larval stage, suggesting their possible roles in digestion or parasitoid–host interaction as salivary proteins (Fig. 4b and Supplementary table S41, Supplementary Material online). Similarly, the expanded Venom allergen family in Parasitoida may also be related to the venom evolution of parasitoid wasps (Fig. 4c and Supplementary fig. S23, Supplementary Material online). And we found 15 parasitoid species have used the members of this family as their venom (Crawford et al. 2008; Vincent et al. 2010; Colinet et al. 2013; Dorémus et al. 2013; Zhu 2016; Zhao et al. 2017; Liu et al. 2018; Alvarado et al. 2020; Becchimanzi et al. 2020; Yang et al. 2020; Gatti et al. 2021; Yang et al. 2021; Ye et al. 2023). Furthermore, the gene family also exhibited diverse gene expression patterns, which suggests that the gene may have multiple functions (Fig. 4d and Supplementary table S42, Supplementary Material online). Other enlarged gene families in Parasitoida that have evidence related to venom components include Neprilysin (Colinet et al. 2014; Ye et al. 2022), Serpin (Yan et al. 2023; Ye et al. 2023; Zhou et al. 2023), and Trypsin (Yu et al. 2023). Overall, our findings indicate that the extensive duplication of certain genes in parasitoid wasps has played a role in the evolution of venom diversity. This may enhance the adaptability of parasitoid wasps to different hosts, thereby contributing to the species diversity in Parasitoida.

We also identified 199 gene families that were missing in a higher proportion of Parasitoida than in other taxa (i.e. the gene family is lost in more than 20% of parasitoids but in no more than 2% of other taxa) (Supplementary table S43, Supplementary Material online). Several losses in these gene families may be associated with the parasitoid lifestyle of species in Parasitoida. For example, we hypothesized that the loss of some gene families/subfamilies associated with detoxification metabolism (such as UDP-glycosyltransferase and Cytochrome P450) may be related to the reduction of toxic substances contained in the hosts of some parasitoids. Eleven gene families with GO terms related to embryo development exhibited a higher loss proportion in parasitoid wasps (Supplementary table S43, Supplementary Material online). This suggests that the early developmental processes of parasitoid wasps may have been altered compared to other hymenopterans, possibly in response to adaptations to parasitic life. In addition, our analysis also recovered our previous finding that Vitellogenin and its receptor (Vitellogenin receptor) have been repeatedly lost in many parasitoid wasps (Zhao et al. 2025). The loss of these genes may result in a reduction or absence of yolk proteins in parasitoid wasp eggs, which is consistent with some phenotypic observations (Ralec 1995; Grbic and Strand 1998; Donnell 2004). It is proposed that this may be related to the fact that parasitoid wasp eggs are able to obtain nutrients from the host.

### Genomic Features of Independent Secondary Phytophagy

**Fig. 5.**
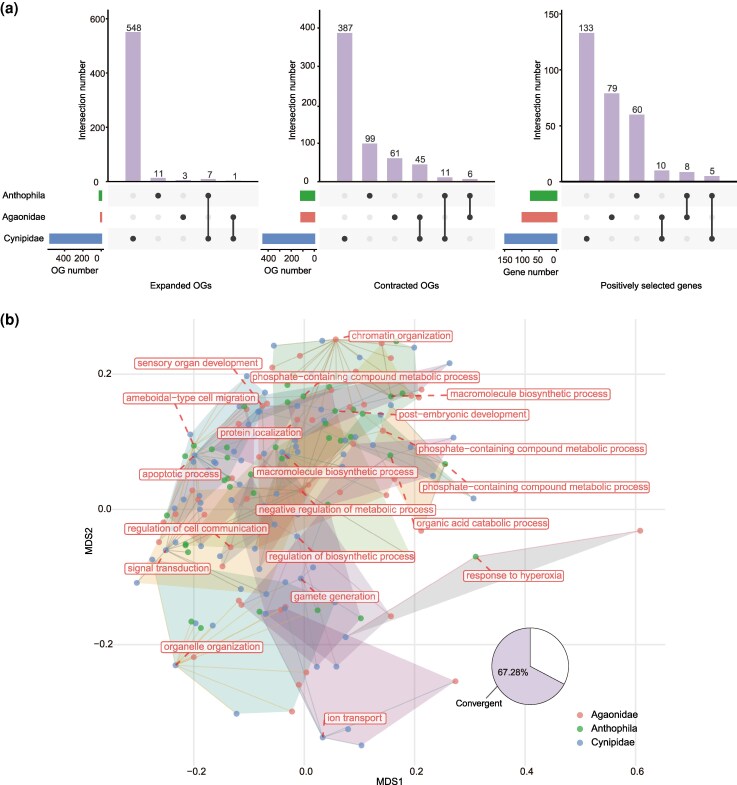
Genomic features of three independent origins of secondary phytophagy. a) Upset charts showing the number of shared and unique expanded, contracted, and positively selected gene families among three clades of independent transitions to secondary phytophagy: Cynipidae, Agaonidae, and Anthophila, with intersection size representing the number of shared OGs in each category. No expanded or contracted OGs, or positively selected genes, were shared by all three groups. b) Functional convergence of exclusively positively selected genes in the three branches of secondary phytophagy origins. Each dot represents an OG to which the exclusively positively selected gene belongs. The centroid OG of each polygon is labeled with potential functions. Of the OGs with exclusively positively selected genes, 67.28% were clustered into 19 functional clusters, indicating functional convergence signals.

During the evolution of Hymenoptera, a shift from phytophagy to parasitoidism occurred at the ancestral node of Orussidae and Apocrita, after which some hymenopterans from different superfamilies re-evolved into phytophagy (i.e. secondary phytophagy). And a recent evolutionary study has proposed that secondary phytophagy in Hymenoptera plays a key role in their species diversity (Blaimer et al. 2023). However, the genomic basis for the hymenopterans that evolved back to phytophagy is still unknown. Moreover, it is worth exploring whether there are any similarities in the genomic features underlying these independent transitions (i.e. convergent or parallel evolution). In the present study, our Hymenoptera phylogeny captured three independent transitions to secondary phytophagy, located at the terminal branch leading to the gall wasp *Belonocnema kinseyi* (Cynipoidea, Cynipidae) (node E in Fig. 2a), the ancestral branch of two fig wasps, *Ceratosolen solmsi* and *Eupristina verticillata* (Chalcidoidea, Agaonidae) (node F), and the ancestral branch of pollen-feeding bees (Apoidea, Anthophila) (node G; Fig. 1). First, we examined the genomic changes, including gene family expansions/contractions and genes with positive selection on these target branches. In general, the genomic changes of these three branches showed a high degree of branch specificity, both in terms of the number of changes and the composition of genes or gene families (Fig. 5a and Supplementary tables S44 to S46, Supplementary Material online). In addition, our analysis did not reveal any instances of gene family expansion, contraction, or positive selection of genes that were common to all three branches (Fig. 5a). This result suggests that the genomic changes behind the three independent transitions to phytophagy in Hymenoptera may be distinct. This may be attributed to differences in the types of plant-derived foods consumed (e.g. plant galls for the gall wasp and pollen for bees). Nevertheless, we did identify some genomic changes that were shared by two branches or specific to one branch, which may be associated with secondary phytophagy (Fig. 2a). For example, we observed that the Maltase family has expanded on the branches related to the secondary phytophagy of both the gall wasp and fig wasps, which probably plays a role in enhancing the carbohydrate metabolism of these species (Supplementary table S44, Supplementary Material online). This is also supported by the expansion of the Glucose dehydrogenase family in the gall wasp *B. kinseyi*. We also noticed the family contractions (e.g. Venom metalloproteinase and Venom allergen) related to venom functions in the gall wasp and fig wasps (Supplementary table S45, Supplementary Material online). This may be indicative of the degradation of the venom components originally used to attack insect hosts following their transformation to phytophagy. In bees, a large expansion of Odorant receptors was observed, probably to improve the ability to recognize and distinguish volatile substances in plants so as to effectively recognize and select food. Additionally, we found the Peritrophin family expanded, which is regarded as the most important physical immune barrier of the gut in arthropods (Hegedus et al. 2009), possibly as an adaptation from sarcophagy to pollen feeding. In contrast, the shrinkage of the Uricase gene family could be an evolutionary response to the lower metabolic capacity for purine degradation (Zhu et al. 2018). In *B. kinseyi*, the expansion of Glutathione S-transferase could also be required for the detoxification of phytochemicals (Gloss et al. 2014; Gloss et al. 2019) (Fig. 2a). Meanwhile, positive selection analysis resulted in 97, 73, and 148 positively selected genes in fig wasps, bees, and the gall wasp, respectively, of which 272 genes were exclusive (Fig. 5a and Supplementary table S46, Supplementary Material online). We further discovered that 67.28% of these exclusively positively selected genes were clustered into 19 functional clusters, exhibiting signals of functional convergence (Fig. 5b and Supplementary table S47, Supplementary Material online). However, no more than 30% of expanded and contracted OGs could be clustered (Supplementary figs S24 and S25, Supplementary Material online and Supplementary tables S48 and S49, Supplementary Material online). Overall, the genomic signatures of dietary changes can be summarized into three functional categories: chemoreception, digestion, and detoxification.

Plants exploit the glucosinolate–myrosinase system to produce toxic hydrolysis products to defend themselves from herbivores (Winde and Wittstock 2011; Lv et al. 2022). In *Athalia rosae* and some aphids, glucosinolate can be rapidly sequestered into the hemolymph, where it is hydrolyzed by myrosinases (Bridges et al. 2002; Müller 2009; Abdalsamee et al. 2014; Oeyen et al. 2020). Thus, a high copy number of the myrosinase gene may characterize phytophagous species (Oeyen et al. 2020). However, in our study, the copy number of myrosinase was indeed high in phytophagous sawflies, but there was no expansion in secondary phytophagous species (Supplementary fig S26, Supplementary Material online). Therefore, we speculate that secondary phytophagous species may employ strategies of detoxification metabolism that are different from those of phytophagous species.

## Conclusion

In this study, our comparative genomic analyses revealed many genomic changes (such as gene family evolution, protein domain evolution, evolutionary rate, and selection) in the evolutionary history of Hymenoptera. We correlated some genomic changes with specific innovations, including the evolution of parasitoidism, wasp-waist, stinger, and secondary phytophagy. In comparing the two major hymenopteran clades, Parasitoida and Aculeata, we uncovered distinct genomic features indicative of their independent evolutionary trajectories, including variations in pathway coverage and gene family dynamics, which may reflect adaptation of their divergent lifestyles (highly parasitoidism vs. complex free-living habits, which are not limited to parasitoidism). Furthermore, we investigated the genomic basis for the hymenopterans that evolved back to phytophagy in three independent lineages. Overall, our study reveals the patterns of genome evolution in Hymenoptera and lays the groundwork for significantly advancing our understanding of the evolutionary trajectories and diversification processes of this group of insects.

## Materials and Methods

### Data Preparation

We collected genomic data on hymenopteran species from public databases, including NCBI, Darwin Tree of Life, and National Genomics Data Center (NGDC) (data acquired before 2023 August 1). Initially, 228 genomes were obtained. These genomes were screened based on two criteria: first, they required high-quality annotation (BUSCO values above 85%); second, to avoid taxonomic bias, we randomly selected three representative genomes for any genus with more than three suitable entries. In total, 131 species were chosen, including 13 superfamilies and 29 families (Supplementary fig. S1, Supplementary Material online and Supplementary table S1, Supplementary Material online). To avoid redundancy, we selected a representative protein isoform (i.e. the isoform with the longest amino acid sequence) for each gene in each species. Assembly quality of each genome was assessed with QUAST v5.2.0 (Gurevich et al. 2013), and completeness was assessed with BUSCO v5.4.7 (Manni et al. 2021) (-m prot -l insecta_odb10). Finally, the average BUSCO score reached 97.26%. For more detailed information about species and public data repositories, see Supplementary table S1, Supplementary Material online. To reduce the impact of potential gene annotation errors, we corrected potential chimeric genes and broken genes. We first used Broccoli v1.2 (Derelle et al. 2020) to identify OGs from all protein sequences of 131 species. Genes assigned to two or more OGs by Broccoli v1.2 were considered chimeric genes. Those that belonged to the same OG were sequenced on the same strand on the same chromosome, showing low sequence similarity (less than 30% calculated using MAFFT v7.310 [Katoh et al. 2002]), and matching different portions of the longest homolog protein in *Nasonia vitripennis* were considered broken genes. Detailed correction procedures for chimeric and broken genes are referenced in a previous study (Mantica et al. 2024).

### Phylogenetic Analysis

To identify OGs from the protein sequences of all 131 species, we performed orthogroup inference using OrthoFinder v2.5.4 (Emms and Kelly 2019). We systematically assessed the impact of the MCL inflation parameter (−*I*; values tested: 1.5, 2.0, 3.0, 4.0) on clustering robustness. Comparative analysis confirmed that *I* = 1.5 yielded the most biologically credible orthogroups (see Supplementary note, Supplementary Material online). In total, 1,002 single-copy genes were identified for species tree inference based on the STAG method that is integrated in OrthoFinder (Emms and Kelly 2018; Emms and Kelly 2019). The protein sequences of these genes were aligned with MAFFT v7.505 (Katoh et al. 2002) and subsequently concatenated into supergenes, which were then trimmed using an OrthoFinder pipeline (see https://github.com/davidemms/OrthoFinder). This alignment was then used to construct a maximum-likelihood phylogenetic tree using IQ-TREE 2.0.3 (Minh et al. 2020) with 1,000 ultrafast bootstraps. At the time, the best-fitting model was estimated by ModelFinder (Kalyaanamoorthy et al. 2017). Species divergence times were estimated with r8s1.81 (Sanderson 2003) using 13 calibrations based on previous studies (Peters et al. 2017). The time points were as follows: Hymenoptera: 281 Mya, Tenthredinoidea: 106 to 176 Mya, Orussoidea + Apocrita: 211 to 289 Mya, Apocrita: 203 to 276 Mya, Ichneumonoidea: 151 to 218 Mya, Chalcidoidea: 105 to 159 Mya, Cynipoidea: 69 to 125 Mya, Aculeata: 160 to 224 Mya, Apoidea: 128 to 182 Mya, Vespoidea: 114 to 180 Mya, Formicidae: 65 to 127 Mya, Apidae: 68 to 99 Mya, and Braconidae: 116 to 177 Mya.

### Gene Family Evolution

We used CAFE5 (Mendes et al. 2021) to determine gene family expansion and contraction for each branch with default parameters, using the gene families detected by OrthoFinder and a species-level phylogeny with divergence times as inputs. Gene families with a *P* value less than 0.05 were considered to have significant expansions and contractions. In order to estimate the differences in the distribution of rapid evolutionary events between Parasitoida and Aculeata, we used Chi-square tests, and if FDR-adjusted *P* < 0.05 and odds ratio > 1, the gene family was considered to have a significantly different occurrence rate of rapid evolutionary events between Parasitoida and Aculeata. The gene gain and loss rate for each clade was calculated as the sum of gene gains and losses for all OGs divided by the divergence time. For each OG, the gene gain and loss rates of different branches were calculated as the count change of OG between it and its parent clade divided by the corresponding divergence time.

### Cuticular Protein Family Analysis

BLASTP analyses were performed against protein sequences of all species using cuticular protein sequences from hymenopteran species (i.e. *Apis mellifera*, *Microplitis mediator*, *P. puparum*, and *N. vitripennis*) as probes (-evalue 1e-5). Meanwhile, the genomes of all species were searched by TBLASTN with the above probes. The domains of proteins aligned with BLASTP and TBLASTN were predicted using hidden Markov model searches with HMMER 3.4 (https://github.com/EddyRivasLab/hmmer) against the Pfam database (http://pfam.xfam.org/). For the cuticular proteins analogous to the peritrophins (CPAP) family, the CBM_14 domain (PF01607) was required, of which the CPAP1 subfamily had one and CPAP3 had three. For the cuticular proteins with Rebers and Riddiford (R&R) consensus (CPRs), the Chitin_bind_4 domain (PF00379) was required, and they were divided into RR-1 and RR-2 subfamilies according to the best hit. The CPF family was identified based on the Cuticle_3 domain (PF11018), and the Tweedle (TWDL) family was identified based on the DUF243 domain (PF03103).

### Selection Pressure Analysis

We selected 27 representative species of the major groups of Hymenoptera and identified 2,060 one-to-one orthologous protein-coding genes using OrthoFinder 2.5.4 (Emms and Kelly 2019); for detailed species, see Supplementary table S50, Supplementary Material online. aBSREL (adaptive Branch-Site Random Effects Likelihood) (Smith et al. 2015) implemented in the HyPhy (Kosakovsky Pond et al. 2020) package was used for detecting positively selected genes at key points in Hymenoptera evolution (FDR-adjusted *P* < 0.05, Bonferroni–Holm correction). In order to compare selection pressure between Parasitoida and Aculeata clades, we first calculated the *d*_N_/*d*_S_ ratio for each branch in the phylogenetic tree using the free-ratio model (model = 1, NSsites = 0) in PAML 4.9 (Yang 2007). We also performed the one-ratio model (model = 0, NSsites = 0) and compared it to the free-ratio model via a likelihood-ratio test to determine if the free-ratio model provided a significantly better fit to the data. Mann–Whitney *U* tests were performed to identify OGs with a significant difference in *d*_N_/*d*_S_ between Parasitoida and Aculeata. To further test *d*_N_/*d*_S_ variation in Parasitoida and Aculeata, we used the CoV, calculated by dividing the standard deviation by the mean of *d*_N_/*d*_S_ in each OG in each group, and compared them using a Mann–Whitney *U* test.

### Protein Domain Analysis

We utilized HMMER 3.3.2 to annotate the protein domains in all species and calculated the number of domain families classified based on PfamA accession. CAFE5 (Mendes et al. 2021) was used to identify significantly accelerated domain families in the same way that gene families were. The domain rearrangements were reconstructed using DomRates (Dohmen et al. 2020) with PfamScan (https://github.com/aziele/pfam_scan) results as inputs.

### GO Annotation and Enrichment Analysis

We annotated proteins from all species using eggNOG-mapper v2 (Cantalapiedra et al. 2021) against the eggNOG5 database. Based on the GO annotation results, we performed GO enrichment analysis of the candidate gene set using GOATOOLS (Klopfenstein et al. 2018) with the go-basic.obo database (http://geneontology.org/). The result of the *P* values for each GO term was corrected for an FDR < 0.05 (Benjamini–Hochberg multitest correction).

### Molecular Evolution Analysis on Functional Categories

The R package constellatoR (https://github.com/MetazoaPhylogenomicsLab/constellatoR) was used to assign GO annotations to each OG and cluster GOs with similar functions using the results of eggNOG-mapper v2 and OrthoFinder as inputs, thus classifying OGs into different functional categories. For each OG in each category, the normalized evolutionary rate was estimated by averaging the ratio of amino acid substitution rates of interspecific homologous proteins to interspecies amino acid substitution rates. PAML 4.9 (Yang 2007) was used to calculate the *d*_N_/*d*_S_ ratio for each OG with the one-ratio model (model = 0 and NSsites = 0) using its respective phylogenetic tree.

### Whole Genome Alignment and Constraint Analysis

We used a 27-way whole genome alignment generated by the reference-free aligner Cactus (Armstrong et al. 2020) and PHAST v1.6 (Hubisz et al. 2011) programs (https://github.com/CshlSiepelLab/phast). We utilized msa_view to retrieve 4-fold degenerate sites based on *B. terrestris* coding gene annotation, and we fitted a phylogenetic model with phyloFit from PHAST v1.6. To quantify the constraint of individual nucleotides, we utilized phyloP (part of the PHAST v1.6 package) to compute the per-base constraint based on the resulting model. Columns with a positive score were considered constrained bases, and those with a negative score were accelerated-evolving bases. Similarly, we used phastCons (Siepel et al. 2005) (part of the PHAST v1.6 package) to find conserved elements. We excluded conserved elements less than 20 bp in length. To further investigate the chromatin accessibility of conserved non-coding elements, we downloaded raw ATAC-Seq data for the egg, larva, pupa, and adult stages of *B. terrestris* (Zhao et al. 2020) from NCBI (accessions in Supplementary table S51, Supplementary Material online). Trimmomatic 0.33 (Bolger et al. 2014) was used to filter raw reads, removing low-quality reads and adapter sequences. Clean reads were mapped to the *B. terrestris* reference genome with Bowtie 2 (Langmead and Salzberg 2012). Then, we used Picard 3.1.1 (https://github.com/broadinstitute/picard) to eliminate PCR duplicates and SAMtools 1.6 (Li et al. 2009) to delete mitochondrial reads. MACS2 (Gaspar 2018) was utilized to identify peaks, and the irreproducible discovery rate (IDR) analysis was performed to assess the reproducibility by comparing the consistency of two biological replicates at the same developmental stage. Peaks with IDR ≤ 0.05 were reproducible across two replicates and were kept for joint analysis with identified conserved noncoding elements.

### Metabolic Pathway Evolution

We used eggNOG-mapper v2 (Cantalapiedra et al. 2021) to perform EC annotations for all 131 hymenopterans, and then ancestral pathways were constructed according to the intersections of annotated ECs in eight sawflies (Tenthredinoidea) and the enzymes of each pathway in KEGG. Pathway coverage was measured as the number of annotated ECs in the pathway divided by the total number of ECs in the constructed ancestral pathway. We considered pathways for which KEGG had a reference pathway for *A. rosae*, and the reconstructed pathway contained at least five ECs. Coverage of each pathway between the Parasitoida and Aculeata species was compared by a Mann–Whitney *U* test, and the FDR-adjusted *P* of less than 0.05 was considered significantly higher or lower. The CoVs of pathway coverage in Parasitoida and Aculeata were calculated as the standard deviation divided by the mean of pathway coverage in these two clades, respectively.

### Functional Convergence Analysis

To investigate the level of functional convergence of interested OGs in three secondary phytophagy transitional ancestral branches, the R package constellatoR was used to calculate the similarity based on the semantic similarity matrix between OGs and cluster them with an exemplar OG. We then used the plotConstellation function to visualize the cluster shared by the interested OGs of the three transition nodes.

### Transcriptome Analysis

Raw transcriptome sequencing data for the second instar larva, third instar larva, fourth instar larva, female pupa, female adult, and venom gland of *A. fulloi* were downloaded from NCBI (accessions in Supplementary table S52, Supplementary Material online). Fastp 0.22.0 (Chen et al. 2018) was used for filtration and quality control, and Salmon v0.14.1 (Patro et al. 2017) was used to calculate the expression level of transcripts (measured as transcripts per million, TPM).

## Supplementary Material

msaf221_Supplementary_Data

## Data Availability

All the data used in this study are listed in Supplementary table S1, Supplementary Material online. The sequence alignment for species tree inference and executed commands and codes has been uploaded to https://github.com/chun-he-316/Hymenoptera-comparative-genomic.
