# Supplementary material for: Large-scale Genome Analyses Provide Insights into Hymenoptera Evolution
Source: Mol Biol Evol. 2025 Sep 12;42(10):msaf221. doi: 10.1093/molbev/msaf221 (PMC12492102; doi:10.1093/molbev/msaf221)
Supplement: msaf221_Supplementary_Data [file msaf221_supplementary_data.zip › Supplementary Notes.pdf]

Supplementary Notes

Large-scale genome analyses provide insights into Hymenoptera evolution

Chun He, Yi Yang, Xianxin Zhao, Junjie Li, Yuting Cai, Lijia Peng, Yuanyuan Liu, Shijiao Xiong, Yang Mei, Zhichao Yan, Jiale Wang, Shan Xiao, Ziwen Teng, Xueke Gao, Hui Xue, Qi Fang, Gongyin Ye, Xinhai Ye

Correspondence:
yexinhai@zafu.edu.cn (X.H. Ye)

This PDF file includes:
Supplementary Note 1

Supplementary Note 1. Orthogroup Clustering Strategy and MCL Inflation Parameter Evaluation

To assess the impact of clustering granularity on gene family inference, we ran OrthoFinder v2.5.4 (Emms and Kelly 2019) using multiple MCL inflation parameters (-I = 1.5, 2.0, 3.0, and 4.0), and compared the resulting orthogroup statistics (Supplementary Notes Table 1). This evaluation allowed us to determine an optimal balance between gene family resolution and cross-species representation.

Supplementary Notes Table 1. Summary of orthogroup clustering metrics across different MCL inflation parameters used in OrthoFinder.

MCL inflation	Total Orthogroups	Single-species Orthogroups	Orthogroups Shared by All Species	O50[1]
1.5	33,045	7,798	1,175	4,254
2.0	40,782	10,810	1,092	4,741
3.0	48,523	14,198	1,064	5,051
4.0	54,022	16,599	1,043	5,220

[1]Number of orthogroups containing 50% of all genes

Analysis revealed that higher inflation parameters increased the total number of orthogroups, singleton counts, and single-species orthogroups, suggesting stricter clustering fragmented gene families into smaller clusters. Excessive fragmentation could artificially inflate lineage-specific gene turnover rates in CAFE5 (Mendes et al. 2021), introducing noise rather than improving accuracy. Concurrently, the number of orthogroups shared by all species decreased as the inflation parameter increased, indicating over-splitting of conserved gene families that are crucial for reliable cross-lineage comparisons and phylogenetic reconstructions. Notably, with -I = 1.5, a relatively small number of orthogroups (O50 = 4,254) accounted for 50% of all genes, indicating that the majority of genes reside in relatively large families. This allows CAFE5 to focus on well-supported, stable gene families for reliable evolutionary inferences.

Furthermore, the same MCL inflation parameter has been utilized in several large-scale comparative genomics studies across diverse taxa, including brown algae (Denoeud et al. 2024), plants (Whiting et al. 2024), and Lepidoptera insects (Han et al. 2024), also demonstrating its robustness and reliability.

Based on this empirical evaluation and precedent in the literature, we retained -I = 1.5 for downstream analyses.

References:

Denoeud F, Godfroy O, Cruaud C, Heesch S, Nehr Z, Tadrent N, Couloux A, Brillet-Guéguen L, Delage L, Mckeown D, et al. Evolutionary genomics of the emergence of brown algae as key components of coastal ecosystems. *Cell*. 2024:187(24):6943–6965.e39. https://doi.org/10.1016/j.cell.2024.10.049.

Emms DM, Kelly S. OrthoFinder: phylogenetic orthology inference for comparative genomics. *Genome Biol*. 2019:20(1):238. https://doi.org/10.1186/s13059-019-1832-y.

Han MJ, Luo C, Hu H, Lin M, Lu K, Shen J, Ren J, Ye Y, Westhof E, Tong X, et al. Multiple independent origins of the female W chromosome in moths and butterflies. *Sci Adv*. 2024:10(25):eadm9851. https://doi.org/10.1126/sciadv.adm9851.

Mendes FK, Vanderpool D, Fulton B, Hahn MW. CAFE 5 models variation in evolutionary rates among gene families. *Bioinformatics*. 2021:36(22-23):5516–5518. https://doi.org/10.1093/bioinformatics/btaa1022.

Whiting JR, Booker TR, Rougeux C, Lind BM, Singh P, Lu M, Huang K, Whitlock MC, Aitken SN, Andrew RL, et al. The genetic architecture of repeated local adaptation to climate in distantly related plants. *Nat Ecol Evol*. 2024:8(10):1933–1947. https://doi.org/10.1038/s41559-024-02514-5.
